# NSC243928 Treatment Induces Anti-Tumor Immune Response in Mouse Mammary Tumor Models

**DOI:** 10.3390/cancers15051468

**Published:** 2023-02-25

**Authors:** Benson Chellakkan Selvanesan, Alvaro de Mingo Pulido, Sheelu Varghese, Deepak Rohila, Daniel Hupalo, Yuriy Gusev, Sara Contente, Matthew D. Wilkerson, Clifton L. Dalgard, Geeta Upadhyay

**Affiliations:** 1 Department of Pathology, Uniformed Services University of the Health Sciences, Bethesda, MD 20814, USA; 2 Henry M. Jackson Foundation for the Advancement of Military Medicine, Bethesda, MD 20817, USA; 3 Moffitt Cancer Center, 12902 Magnolia Drive, Tampa, FL 33612, USA; 4 Department of Anatomy, Physiology, and Genetics, Center for Military Precision Health, Uniformed Services University of the Health Sciences, Bethesda, MD 20814, USA; 5 Innovation Center for Biomedical Informatics, Department of Oncology, Georgetown University Medical Center, Washington, DC 20057, USA; 6 John P. Murtha Cancer Center, Bethesda, MD 20814, USA

**Keywords:** LY6K, mammary tumor model, NKT cells, B1 cells, myeloid derived suppressor cells, NSC243928, 4T1, E0771

## Abstract

**Simple Summary:**

This study used two different syngeneic mouse mammary tumor models to determine the effect of a small molecule NSC243928 on intra-tumoral immune cells. We observed that NSC243928 treatment reduced the tumor burden in vivo and altered the wide range of immune cell infiltration in both models. These results pave the path for further study of the role of NSC243928 in immuno-oncology drug development for triple-negative breast cancer.

**Abstract:**

NSC243928 induces cell death in triple-negative breast cancer cells in a LY6K-dependent manner. NSC243928 has been reported as an anti-cancer agent in the NCI small molecule library. The molecular mechanism of NSC243928 as an anti-cancer agent in the treatment of tumor growth in the syngeneic mouse model has not been established. With the success of immunotherapies, novel anti-cancer drugs that may elicit an anti-tumor immune response are of high interest in the development of novel drugs to treat solid cancer. Thus, we focused on studying whether NSC243928 may elicit an anti-tumor immune response in the in vivo mammary tumor models of 4T1 and E0771. We observed that NSC243928 induced immunogenic cell death in 4T1 and E0771 cells. Furthermore, NSC243928 mounted an anti-tumor immune response by increasing immune cells such as patrolling monocytes, NKT cells, B1 cells, and decreasing PMN MDSCs in vivo. Further studies are required to understand the exact mechanism of NSC243928 action in inducing an anti-tumor immune response in vivo, which can be used to determine a molecular signature associated with NSC243928 efficacy. NSC243928 may be a good target for future immuno-oncology drug development for breast cancer.

## 1. Introduction

Lymphocyte antigen 6K (LY6K), a cancer-testis protein, is highly expressed in 70% of clinical cases of triple-negative breast cancer and the expression of LY6K is associated with poor survival outcome in breast cancers [1]. NSC243928 is part of the NCI small molecule library, which is composed of 2000 anti-cancer molecules (https://dtp.cancer.gov/, accessed on 4 December 2022). We identified that small molecule NSC243928 binds with LY6K specifically [2]. NSC243928 was first identified as a compound with anticancer properties in leukemic models in 1979 [3] and was shown to be effective in inducing cell death in ovarian spheroid cultures in vitro [4]. We discovered that NSC243928 induces cell death in multiple triple-negative cancer cell lines that express high levels of the LY6K protein [2]. We observed that the downregulation of LY6K using shRNA can reduce in vivo tumor growth via signaling pathways associated with immune pathways [5]. A precise mechanism of NSC243928 in cancer cell death is not yet known. Thus, we wanted to see whether a pharmacological agent that binds with LY6K to induce cell death in vitro could also inhibit tumor cell growth in vivo, and whether this inhibition is accompanied by changes in the tumor microenvironment in the context of immune cell infiltration.

To test whether NSC243928, a binder of LY6K, may reduce in vivo tumor growth, we selected two immune-competent syngeneic mammary tumor models, 4T1 and E0771, both models that are well used in immuno-oncology drug development. The 4T1 model, a triple negative mammary tumor model, originates from Balb/c mice, and the E0771 model, a luminal B mammary tumor model, originates from C57BL6 mice. Since the models are available as syngeneic mouse models, they offer a unique opportunity to test the effect of novel therapies on immune cells relevant to sustained tumor growth [6,7]. Here, we tested whether treatment with NSC243928 could induce an anti-tumor immune response in these two mammary tumor models in vivo. We found that NSC243928 could indeed induce immunogenic cell death in the 4T1 and E0771 cell lines in vitro and induce an anti-tumor immune response in vivo, as seen by the immunophenotyping of tumor isografts from the control and treated mice. The analysis of the bulk RNA sequencing supports these findings. These data suggest that the NSC243928 small molecule is a valid anti-cancer agent that can be used to develop novel targeted therapeutics that can mount an effective anti-tumor response in triple-negative breast cancer.

## 2. Materials and Methods

### 2.1. Cells

E0771 and 4T1 cells were obtained from American Type Culture Collection (ATCC), Manassas, VA, USA. The cells were cultured in DMEM supplemented with 10% fetal bovine serum (FBS), 2 mM glutamine, 1× non-essential amino acids, 1 mM sodium pyruvate, and 100 U/mL penicillin/streptomycin, henceforth referred to as DMEM complete medium. All cell culture reagents were purchased from Thermo Fisher Scientific, Invitrogen Corporation, Waltham, MA, USA.

### 2.2. Calreticulin (CRT) Cell Surface Expression

Cells were seeded overnight in DMEM complete medium as described in Section 2.1. Cells were serum starved for four hours before treatment with the indicated drugs for 24 h and followed by flow cytometry analysis for the cell surface expression of APC-CRT (Novus Biologicals, Centennial, CO, USA). A live–dead zombie dye (Thermo Fisher Scientific, Invitrogen Corporation, Waltham, MA, USA) was used to discriminate between the live and dead cells. Cells were labeled as per the manufacturer’s protocol and gated on live cells for the cell surface expression of CRT using a CytoFLEX flow cytometer (Beckman Coulter Life Sciences, Indianapolis, IN, USA). The flow cytometry data were analyzed using FLOWJO software (Becton, Dickinson and Company, Ashland, OR, USA).

### 2.3. HMGB1 Release Assay

Cells were seeded overnight before treatment in DMEM supplemented with 1× insulin-transferrin-selenium (ITS-G) (100×), 2 mM glutamine, 1× non-essential amino acids, 1 mM sodium pyruvate, and 100 U/mL pen/strep. All cell culture reagents were purchased from Thermo Fisher Scientific, Invitrogen Corporation, Waltham, MA, USA. Cells were treated with the indicated drugs for 48 h and conditioned medium (CM) was collected. The CM was centrifuged for 10 min at 3000 rpm to ensure the removal of the cell debris. The CM was subjected to protein precipitation using acetone (Sigma-Aldrich, Inc. St. Louis, MO, USA). For acetone precipitation of the proteins, four times the sample volume of cold (−20 °C) acetone was added to the CM. Precipitation was allowed for 1 h in −20 °C and the CM was centrifuged for 10 min at 13,000–15,000× *g*. The precipitated protein was resuspended in 1× RIPA buffer (Cat # 20–188, Sigma-Aldrich, Inc. St. Louis, MO, USA) and protein was quantified using the Pierce™ BCA Protein Assay Kit (Thermo Fisher Scientific, Invitrogen Corporation, Waltham, MA, USA) according to the manufacturer’s protocol. A total of 50 mg of protein from each sample was separated on a 4–12% SDS-PAGE gel and Western blotting was conducted using a rabbit polyclonal HMGB1 antibody (Novus Biologicals, Centennial, CO, USA) and the bands were visualized using HRP conjugated anti-rabbit IgG (Cell signaling Technology, Danvers, MA, USA). The chemiluminescence substrate was used to detect the signals on an iBright Imaging System (Thermo Fisher Scientific, Invitrogen Corporation, Waltham, MA, USA). Equal loading of the protein was ensured by staining the transferred protein on the membrane with Ponceau S (Thermo Fisher Scientific, Invitrogen Corporation, Waltham, MA, USA) staining prior to developing the Western blot for HMGB1 proteins in the CM.

### 2.4. ATP Assay for Extracellular ATP Release and Cell Viability

Cells were seeded overnight in DMEM complete medium, as described in Section 2.1. Cells were serum starved for four hours before treatment with indicated drugs and intervals. Extracellular ATP release was measured using a RealTime-Glo™ Extracellular ATP Assay (Cat #GA5011, Promega Corporations, Madison, WI, USA) according to the manufacturer’s instructions and the luminescence was recorded on a Promega™ GloMax^®^ microplate plate reader (Promega Corporations, Madison, WI, USA).

The cell viability was monitored by the CellTiter-Glo^®^ Luminescent Cell Viability Assay (Cat #G7570, Promega Corporations, Madison, WI, USA), which measures intracellular ATP as a direct measure of cellular health or metabolic activity. The assay was carried out according to the manufacturer’s instructions and the luminescence was recorded on a Promega™ GloMax^®^ microplate plate reader (Promega Corporations, Madison, WI, USA).

### 2.5. Isograft Mouse Model, Tumor Measurements, and Treatment

All animal experiments were approved by The Uniformed Services University of the Health Sciences Institutional Animal Care and Use Committee (PAT-21-060). The 5 × 10^5^ E0771 or 4T1 cells were injected subcutaneously into the ventral abdominal mammary chain of C57BL6 or Balbc 5 to 8-week-old female mice (Charles River Laboratories, Wilmington, DE). Five mice per group for E0771 and ten mice per group in 4T1 were used. Tumors were measured using Vernier calipers by measuring the width (W) and length (L). The length was considered along the body axis. Tumor volumes (V) were calculated using the formula V = (W^2^ × L)/2 [8]. The tumor growth rate was calculated as volume per day as described [9]. Tumor isografts were grown to be larger than 50 mm^3^, followed by drug treatment. Mice were treated with 50 mg/kg of NSC243928 via IV route the first time, the IP route the second time, and the IV route was used the third time. As per guidance from the IACUC, an IP route was used in between IV injections to minimize distress to the animal tail. Mice were euthanized after the indicated treatments to harvest the tumor tissue.

### 2.6. Blood Collection and MDSC Analysis

Blood was collected by cardiac puncture in a terminal procedure. ACD solution (Sigma Aldrich, St. Louis, MO, USA) was added to prevent the coagulation. Red blood cells were lysed using ACK lysis buffer (Thermo Fisher Scientific, Invitrogen Corporation, Waltham, MA, USA) [10]. Next, the cells were stained with live/dead viability dye (Zombie Yellow, BioLegend, San Diego, CA, USA), followed by Fc block and stained with APC tagged CD11b and APC-Cy7 tagged Gr-1 (Ly-6G/Ly-6C), the double positive cells were taken as MDSC. The cells were analyzed using a CytoFLEX flow cytometer (Beckman Coulter Life Sciences, Indianapolis, IN, USA). The data were analyzed using FlowJo software, version 10.8.1 (Becton, Dickinson and Company, Ashland, OR, USA).

### 2.7. Immunophenotyping of the Isografts

**Figure 1:**
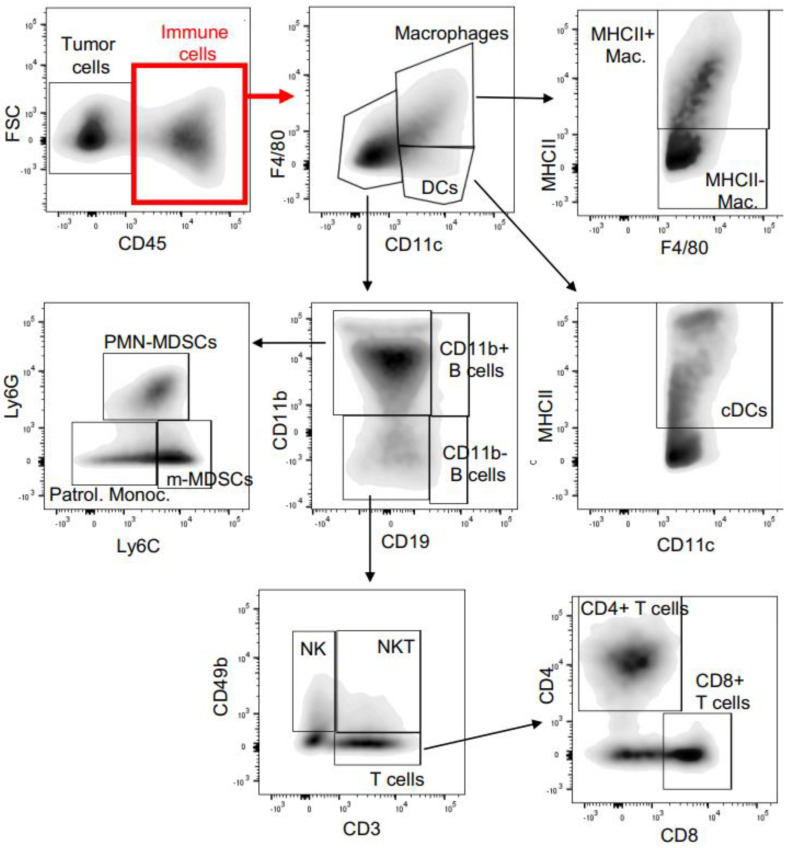
Gating strategy. The single cell suspension was subjected to live dead staining and live CD45 cells were gated for further analysis. The gated cells are shown in a density plot with outliers on a grey scale. The dark grey (black) pockets of the plot represent the main population of the gated cells. The grey represents the outlier cells. Individual cell populations and associated markers are shown in the panels.

Tumor isografts were collected in RPMI medium (Thermo Fisher Scientific, Invitrogen Corporation, Waltham, MA, USA). Tissue was enzymatically dissociated in collagenase solution containing 1.5 mg/mL collagenase IV (Thermo Fisher Scientific, Invitrogen Corporation, Waltham, MA, USA) and DNase solution at 0.1 mg/mL (Roche Holdings, Basel, Switzerland) at 37 °C for 20 min, filtered through a 70-micron filter, and divided into three parts for immunostaining with antibodies in a myeloid panel, lymphoid panel, and in vitro stimulation with PMA/ionomycin and staining to analyze the T-cell activation. The myeloid and lymphoid panel were composed of the following antibodies; BV711 tagged CD45, BV421 tagged CD3, BUV737 tagged CD19, APV-R700 tagged CD11b, BV650 tagged CD11c, APC tagged F4/80, PE-Cy7 tagged MHC II, PerCP-Cy5 tagged Ly6C, BUV395 tagged Ly6G, AF488 tagged CD49b, PE tagged CD103, PE-Dazzle tagged CD4, and BV785 tagged CD8 from BioLegend, San Diego, CA. The single cells subjected to PMA/ionomycin stimulation were analyzed for T-cell activation using the following antibodies: BV711 tagged CD45, BV421 tagged CD3, BUV395 tagged CD4, AF647 tagged CD8, AF488 tagged CD107, BV650 tagged IFNγ, and PE-CY7 tagged TNFα from BioLegend, San Diego, CA. The live CD45 cells were gated for the quantification of various immune cell populations (Figure 1). An equal number of CD45 cells was selected to compare the cell population in the control vs. the treated isografts to remove the tumor size bias from the study. The flow cytometry was performed on a BD LSRII flow cytometer and the flow cytometry data were analyzed using FLOWJO software (Becton, Dickinson and Company, Ashland, OR, USA).

### 2.8. Total RNA Sequencing and Data Analysis

Total RNA was isolated using the RNeasy Kit and subjected to on-column DNA digest (Qiagen Inc., Germantown, MD, USA). The RNA was quantified using the Quant-IT RiboGreen RNA Reagent (Thermo Scientific, Waltham, MA, USA) and measured with a Spectramax Gemini XPS plate reader (Molecular Devices, San Jose, CA, USA). RNA integrity was assessed using automated capillary electrophoresis on a Fragment Analyzer (Advanced Analytical Technologies Inc., Ankeny, IA, USA) with samples passing quality control for RIN values >9. A total RNA input of 200 ng was ideally used for library preparation using the TruSeq Stranded mRNA Library Preparation Kit (Illumina, San Diego CA, USA). Sequencing libraries were sequenced on a NovaSeq sequencer (Illumina, San Diego CA, USA). Paired-end reads were aligned to the mouse reference genome (mm10) using MapSplice (version v2.2.2) with fusion transcript detection enabled [11]. Gene read counts against GENCODE (version 28) “basic” gene models were calculated by HTSeq (version 0.9.1) with parameters: -s reverse -t exon -m intersection-nonempty [12]. Multi-mapping reads were discarded from the analysis. Sample read counts per gene were then normalized using EdgeR by log2 transformation with a minimum CPM of 0.5 [13]. Missing values within the cohort were imputed using the gene median value. Differential expression as performed between each case and control pair using DESeq2 with a FDR cutoff of 0.1, and a minimum fold change of 2 [14]. The most differentially expressed genes were further narrowed down using a fold change cutoff of at least 2 and FDR cutoff of 0.05. The intersection was taken to identify the genes commonly differentially expressed between all experiments and cell lines. The list of differentially expressed genes was used for pathway enrichment analysis using Pathway Studio.

### 2.9. Immune Cell Compositions of 4T1 and E0771 Isografts

Paired-end reads were analyzed using the Nextflow nf-core/rnaseq pipeline [15]. Read count matrices for the control and treated isografts of each model were extracted from Star-Salmon quant.sf files and analyzed on the ImmuCC server using the local linear semi-supervised regression method. This method has been validated to predict mouse immune cell composition in RNA-Seq data [16]. The Wilcoxon rank sum test was used for statistical analysis using the RStudio program. The data were considered significant with *p* < 0.05 with a confidence interval not including 1 or 0 [17].

## 3. Results

### 3.1. NSC243928 Induces Immunogenic Cell Death in the E0771 and 4T1 Cell Lines

Previously, we reported that NSC243928 induces cell death in multiple triple-negative breast cancer cell lines [2]. However, the molecular mechanism of cell death was not explored. Cell death, known as immunogenic cell death (ICD), can induce an anti-tumor immune response in vivo [18]. ICD is characterized by the exposure of calreticulin (CRT) on the cell surface and the extracellular release of high mobility group box 1 (HMGB1) and ATP [19,20].

**Figure 2:**
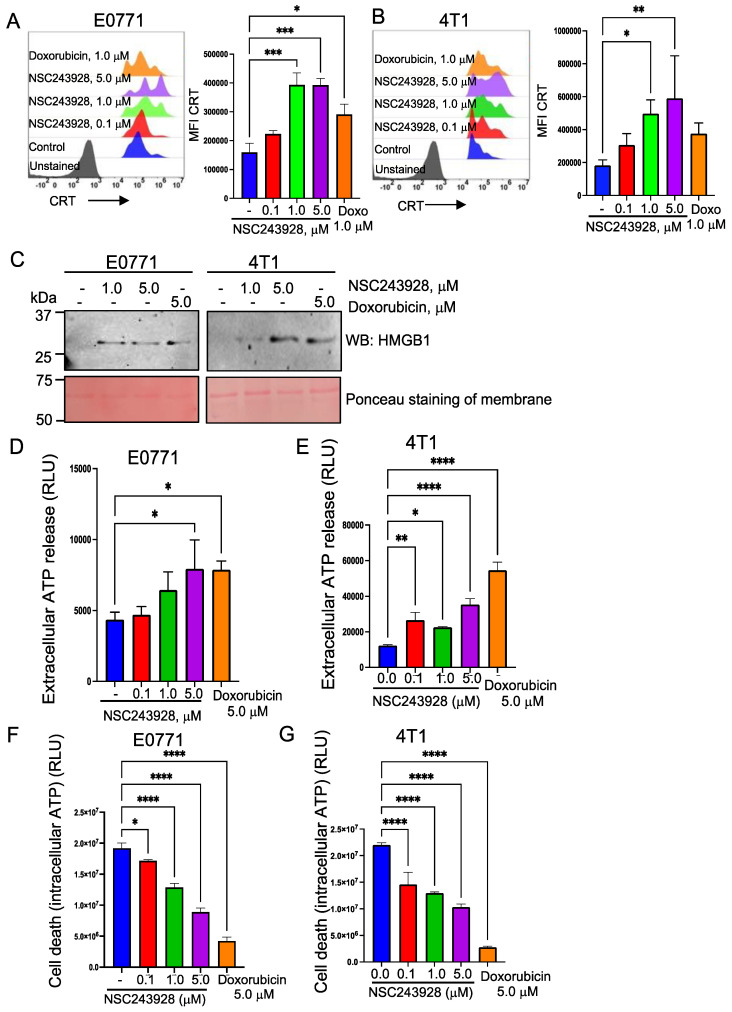
NSC243928 increases immunogenic cell death (ICD). (**A**) E0771 cells and (**B**) 4T1 cells were treated with the indicated concentration of NSC243928 and doxorubicin was used as the appositive control for ICD). (**C**) Treatment with NSC243928 led to an increased release of the HMGB1 protein in the conditioned medium (CM). Ponceau staining of the blot showed equal loading based on the unidentified abundant protein present in CM. Original blot see Supplementary File S1. (**D**) E0771 cells and (**E**) 4T1 cells showed an increased release of ATP in the conditioned medium upon the indicated drug treatments. (**F**) E0771 cells and (**G**) 4T1 cells showed increased cell death, as measured by intracellular ATP levels upon the indicated drug treatments. RLU—renila luciferase units. All experiments were performed at least three times independently. GraphPad Prism software was used for statistical analysis using ordinary one-way ANOVA multiple comparison and the Fisher LSD test. * *p* < 0.05 was considered significant, ** *p* < 0.005, *** *p* < 0.0005, **** *p*<0.0001.

To test whether NSC243928 can directly facilitate the cell surface exposure of CRT, E0771 and 4T1 cells were treated with increasing concentrations of NSC243928. We used doxorubicin as a positive control that has been shown to induce ICD [21]. We observed that the cell surface expression of CRT in the live cells was increased in the E0771 and 4T1 cells in a dose dependent manner (Figure 2A,B). The treatment with NSC243928 led to the release of the HMGB1 protein (Figure 2C) and increased levels of extracellular ATP (Figure 2D,E) in the E0771 and 4T1 cell lines. NSC243928 induced cell death at these concentrations (Figure 2F,G).

### 3.2. NSC243928 Reduces the Tumor Growth In Vivo and Induces a Systemic Anti-Tumor Immune Response

**Figure 3:**
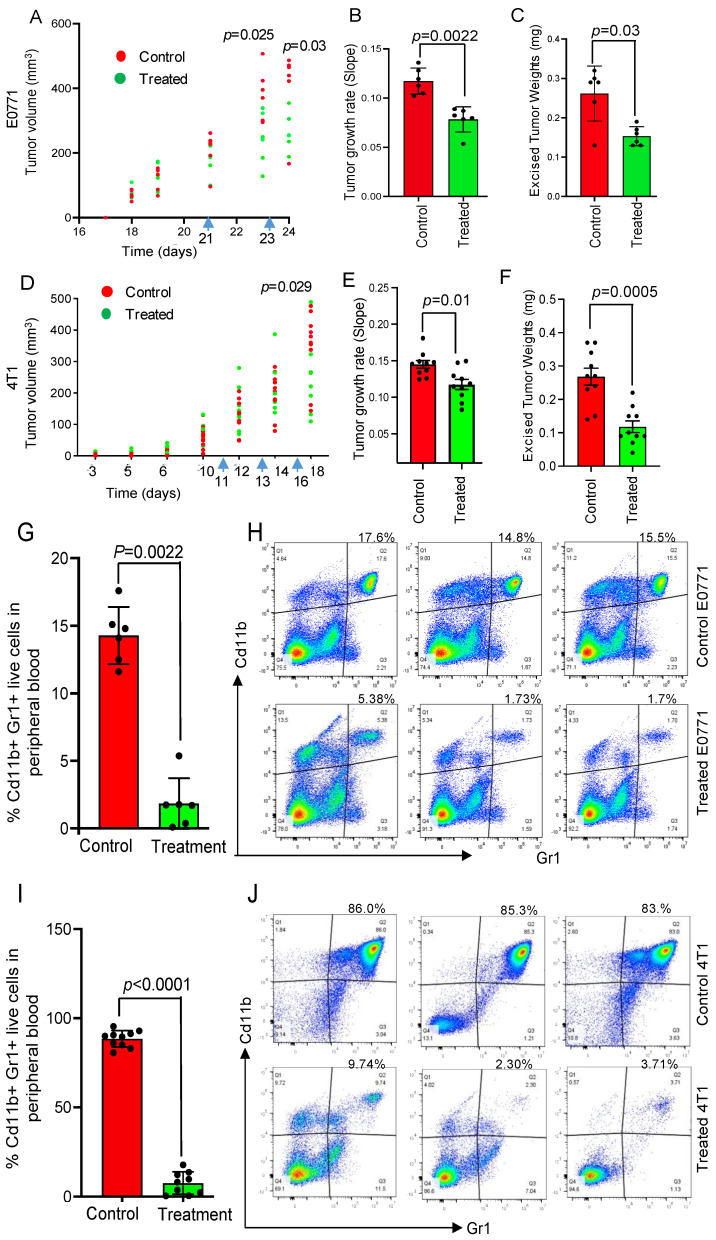
Effect of NSC243928 on the in vivo tumor growth and induction of immunosuppression in mouse mammary tumor models. (**A**) Tumor isografts in E0771 were grown to be larger than 50 mm^3^. Mice were treated with 50 mg/kg of NSC243928, first doses via IV and second dose via IP. (**B**) E0771 mammary tumor growth was significantly reduced in only two doses of the NSC243928 treatment. (**C**) Excised tumor weights were significantly reduced in the NSC243928 treated E0771 group. (**D**) Tumor isografts in 4T1 were grown to be larger than 50 mm^3^. Mice were treated with 50 mg/kg of NSC243928, first dose via IV, second dose via IP, and third dose with IV. (**E**) 4T1 mammary tumor growth was significantly reduced after three doses of the NSC243928 treatment. (**F**) Excised tumor weights were significantly reduced in the NSC243928 treated 4T1 group. (**G**–**J**) Peripheral blood was collected at the time of euthanasia. Live dead zombie dye was used to discard dead cells from the flow cytometry analysis. CD11b and Gr1 labeling were performed to identify the population of MDSCs in the E0771 and 4T1 model as indicated. The upper right quadrant represents CD11b^+^Gr1^+^ cells and the percentage is shown. Statistical analysis was performed with the non-parametric *t*-test Mann–Whitney Test using GraphPad Prism software. *p* < 0.05 was considered significant.

Induction of ICD is linked to the activation of the immune system in the in vivo tumor microenvironment [20]. Therefore, we tested the effect of NSC243928 treatment in vivo. We observed an immediate significant reduction in tumor growth rate and weight following two treatments in E0771 and three treatments in the 4T1 model (Figure 3A–F). These data are also described in a separate study focused on the non-immunogenic effects of NSC243928 (manuscript in preparation). Myeloid derived suppressor cells (MDSCs), defined by positive labeling of CD11b and Gr1 antibodies, are major tumor immune suppressor cells [10]. We found that peripheral MDSCs (CD11b^+^Gr1^+^) were significantly downregulated in drug-treated E0771 and 4T1 tumor models (Figure 3G–J).

### 3.3. Transcriptome Analysis Revealed That NSC243928 Induces an Immune Responsive Tumor Microenvironment

Systemic downregulation of MDSCs upon drug treatment indicated that treatment with NSC243928 was able to trigger an anti-tumor immune response in vivo. Thus, we looked into differential gene expression analysis to identify a broad transcriptional effect due to NSC243928 treatment in vivo. We found that a total of 228 unique genes were differentially expressed in the NSC243928 treated E0771 isograft (Table S1), and 372 unique genes were differentially expressed in the NSC243928 treated 4T1 isograft (Table S2), while 89 genes were common to the NSC243928 treated E0771 and 4T1 isografts (Table S3). Pathway Studio analysis of the differentially expressed gene list revealed that pathways associated with immune regulation and oncogenic signaling were altered in drug treated isografts (Table 1).

**Figure 4:**
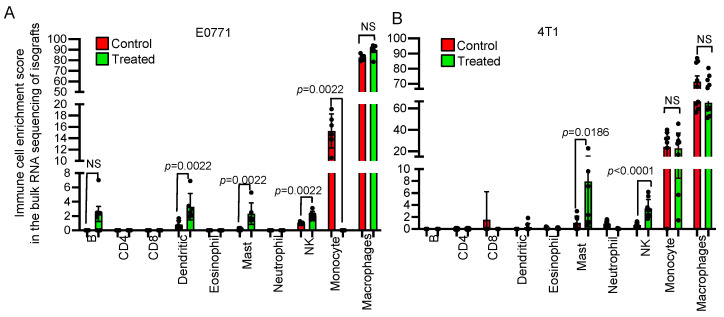
Immune cell enrichment analysis of the RNA-Seq data using the seq-ImmuCC webtool. (**A**) E0771 and (**B**) 4T1 treated isografts vs. the control isografts.

We used an online bioinformatic tool, the seq-ImmuCC program, which uses RNA-Seq data to predict the distribution of a pre-defined set of immune cell populations [16]. NSC243928 treated E0771 isografts showed increases in B-cells, dendritic cells, mast cells, NK cells, and a decrease in the monocyte population (Figure 4A). NSC243928 treated 4T1 isografts showed an increase in mast and NK cells (Figure 4B).

### 3.4. Tumor Infiltrating Lymphocytes Show a Distinct Pattern upon NSC243928 Treatment in the E0771 and 4T1 Models

**Figure 5:**
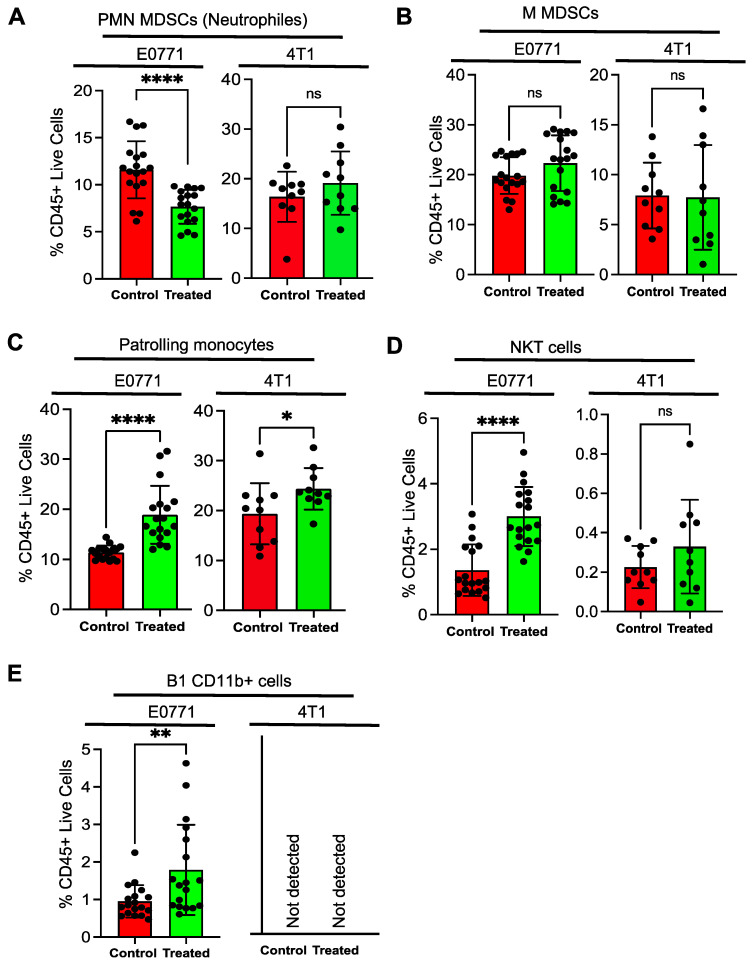
Analysis of tumor infiltrating lymphocytes from tumor isografts. Single cell isolation was performed with enzymatic dissociation. Cells were stained with live dead dye and fluorescent tag antibodies (detailed in the method section) used to identify and quantitate cell populations of (**A**) PMN^−^MDSC (CD11b^+^Ly6G^+^Ly6C^low^), (**B**) M-MDSC (CD11b^+^Ly6C^+^/Ly6G ^low−^), (**C**) Patrolling Monocytes (CD11b^+^, Ly6C^low^, Ly6G^−^), (**D**) NKT cells (CD3^+^, CD49b^+^, CD11b^−^), (**E**) B1 cells (MHCII^+^, CD19^+^, CD11b^+^). * *p* < 0.05 was considered significant, ** *p* < 0.005, **** *p*<0.0001.

Tumor isografts were subjected to single cell dissociation and analyzed for the presence of intra^−^tumoral immune cell populations. Immune cell populations with a distinct phenotype were identified with specific markers using the gating strategy described in Figure 1. Equal numbers of live CD45 cells were used for the quantitative intra-tumoral immune cell populations in the control and treated isografts to remove the isograft bias size. Peripheral MDSCs were identified using CD11b^+^ and GR1^+^ markers. The Gr1 marker is a composite epitope between the Ly6C and Ly6G antigens. MDSCs can be further subdivided into granulocytes or polymorphonuclear MDSCs (PMN MDSCs) identified by CD11b^+^Ly6G^+^Ly6C^low^ cells and monocytic MDSCs (mMDSCs) identified by CD11b^+^Ly6C^+^/Ly6G^−^ cells. PMN MDSCs are terminally differentiated MDSCs, which reside in the tumor microenvironment and suppress the antitumor immune response [38,39]. We observed that the PMN-MDSC cells were significantly downregulated in the NSC243928 treated E0771 model but not in the 4T1 model (Figure 5A). We observed that the tumor residing M-MDSC populations were not significantly altered by the NSC243928 treatment of isografts in either model (Figure 5B). Patrolling monocytes (CD11b^+^, Ly6C^low^, Ly6G^−^) are associated with anti-tumor immune response [40]. Patrolling monocyte levels were significantly increased by NSC243928 treatment in both the E0771 and 4T1 models (Figure 5C). NKT (CD3^+^, CD49b^+^) cells were significantly increased in the NSC243928 treated tumor isografts from E0771 but not in the 4T1 model (Figure 5D). A subpopulation of B^−^ cells, namely B1 cells (MHCII^+^, CD19^+^, CD11b^+^+), which are important for anti-tumor immune response [41], was significantly increased in the NSC243928 treated tumor isografts from the E0771 model (Figure 5E).

NK cells (CD3^−^, CD49b^+^) showed an upward trend but did not reach significance in the NSC243928 treated mice from both models (Figure S1A). The total B-cell (MHCII^+^, CD19^+^) levels were not altered in the NSC243928 treated tumor isografts from the E0771 model and were not detected in the NSC243928 treated tumor isografts from the 4T1 model (Figure S1B). The total T-cell (CD3^+^, CD11b^−^) levels were not altered in the NSC243928 treated tumor isografts from the E0771 and 4T1 models (Figure S1C). MHCII^−^TAMS (F4/80^+^ MHCII^−^, CD11b^+^) were not significantly altered in the NSC243928 treated tumor isografts from E0771 and were found to be slightly elevated in the 4T1 model (Figure S1D). MHCII^+^TAMS (F4/80^+^ MHCII^+^, CD11b^+^) was reduced in the NSC243928 treated tumor isografts from the E0771 and 4T1 models (Figure S1E).

### 3.5. Immune Cells from NSC243928 Treated E0771 Tumor Isografts Generate a Better Response in Cytokine Production Ex Vivo Compared to the 4T1 Model

**Figure 6:**
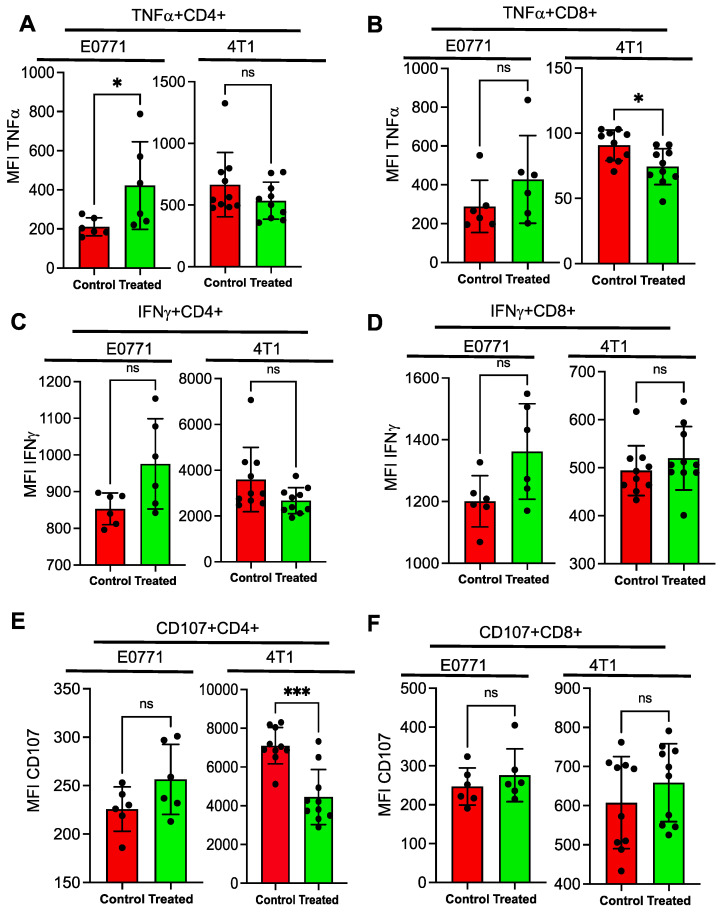
Stimulation of the tumor infiltrating lymphocytes from the tumor isografts. Single cell isolation was performed with enzymatic dissociation. Cells were stimulated with PMA and ionomycin for 4 h. Cells were stained with live dead dye and fluorescent tag antibodies (detailed in the method section) to identify and quantitate the cell population of (**A**) TFNα^+^CD4^+^, (**B**) TFNα^+^CD8^+^, (**C**) IFNγ^+^CD4^+^, (**D**) IFNγ^+^CD8^+^, (**E**) CD107^+^CD4^+^, and (**F**) CD107^+^CD8^+^. The bar graph represents the total cytokine production as observed by the mean fluorescence intensity of the cytokine on the double positive cells. * *p* < 0.05, *** *p* < 0.0005.

Flow cytometry analysis revealed that many immune cell types involved in anti-tumor response such as PMN^−^MDSCs, patrolling monocytes, and NKT cells were increased in the drug treated isografts. Anti-tumor immune cells are known to produce an array of cytokines such as TNFα, IFNγ, and CD107 as a measure of their activity [42]. To assess whether the immune cells that infiltrated the tumor microenvironment in the drug-treated isografts have the capability to produce cytokines, the single cell suspensions from the control and NSC243928 treated tumor isografts were subjected to an ex vivo treatment with PMA and ionomycin for 4 h before flow cytometry analysis for TNFα, IFNγ, and CD107(LAMP1) on the CD4 and CD8 positive (+) T cells. We observed increased levels of TNFα on CD4^+^ cells in the NSC243928-treated E0771 isografts but not from the 4T1 isografts (Figure 6A). Similarly, we observed a trend of increased TNFα producing CD8^+^ cells in the NSC243928-treated E0771 isografts but not in the 4T1 isografts (Figure 6B). Increased IFNγ production in the CD4^+^ and CD8^+^ cells was observed, but it did not reach significance in the NSC243928 treated E0771 isografts (Figure 6C,D). Intra-tumoral immune cells from NSC243928 treated E0771 isografts but not from 4T1 isografts showed a trend of a higher stimulation of CD107(LAMP1) CD4^+^ cells, but it did not reach significance (Figure 6E,F). These data indicate that CD4^+^ and CD8^+^ T^−^cells from the NSC243928-treated E0771 isografts were able to generate increased cytokine production compared to the NSC243928-treated 4T1 isografts.

## 4. Discussion

Breast cancer is one of the malignancies still to see the benefits of immunotherapy advances [43]. Mammary tumor E0771 and 4T1 syngeneic mouse models have been implemented to study drug efficacy including novel immune-oncology drugs for human luminal B, Her2 positive, and stage IV triple negative breast cancer [6,7,44,45,46,47,48]. Thus, we selected these models to determine whether NSC243928 may suppress tumor growth and induce an anti-tumor immune response. Previously, we observed that NSC243928 induced cell death in cancer cells. Here, we showed that NSC243928 was able to induce a specific form of cell death known as immunogenic cell death (ICD) in E0771 and 4T1. Although the precise mechanisms of NSC243928induced ICD remain to be discovered, we were able to show that NSC243928 induced a tumor reduction and anti-tumor immune response in both models.

It was shown that TGFβ signaling associated with fibrotic regulation of the extracellular matrix requires CRT expression, a hallmark of ICD, during ER stress [49]. We previously showed that LY6K is required for canonical TGFβ/Smad signaling, but the effect is still to be determined in fibrotic TGFβ signaling, leading directly or indirectly to ICD. This line of investigation will be pursued in the future to determine whether the NSC243928 effect on TGFβ signaling is associated with ICD response in vitro and in vivo.

We observed that the functional effect of ICD manifested as systemic anti-tumor immune response in drug treated isografts. NSC243928 treatment led to reduced MDSCs (CD11b+ Gr1+) in the peripheral blood, which supports the anti-tumor immune effects of NSC243928 in vivo. MDSCs (CD11b+ Gr1+) can differentiate into PMN MDSCs (CD11b+Ly6G+Ly6C^low^) and M MDSCs (CD11b+Ly6G+Ly6C^−^) in the tumor microenvironment [38]. PMN MDSCs are terminally differentiated cells that have potent immunosuppressive function leading to sustained tumor growth [50,51]. We observed that NSC243928 treatment significantly decreased intra-tumoral PMN MDSCs in E0771 model but not in the 4T1 model. It is plausible that NSC243928 treatment modulates the tumor microenvironment specific to E0771 resulting in suppression of pro-tumorigenic PMN^−−^MDSCs.

Monocytes are important immune cell components, with the classical monocytes having tumor promoting action and the non^−^classical, or patrolling monocytes with CD11b^+^, Ly6C^low^, Ly6G^−^ phenotype having potent anti-cancer and anti-metastatic properties [40]. They help to recruit and activate NK cells and play an important role in immunosurveillance [52,53,54]. NSC243928 treatment led to significantly increased patrolling monocytes in both models, suggesting that this can be an important pathway mechanism associated with the in vivo action of NSC243928. NKT and B1 (MHCII^+^, CD19^+^, CD11b^+^) cells are of emerging interest in the immune-oncology field as these cells have the potential to mount a direct cancer cell death response, so they are part of the adaptive immune response and they have an innate immune response component [41,55]. The NKT cells were significantly increased in the drug treated E0771 model; this was also increased in the drug treated 4T1 model, but the level of significance was not reached. B1 cells were not detected in the 4T1 model. These data indicate that the E0771 mammary tumor model may have a robust anti-tumor response upon NSC243928 treatment. We did not observe the expected effects of NSC243928 treatment on the tumor associated macrophages (TAMs) (F4/80^+^ MHCII^+^, CD11B^+^, CD11c int.) that are associated with the tumor microenvironment [56,57].

Cytokine release from immune cells in response to activation stimuli is considered as a surrogate marker for their activity. To test the activity of immune cells, the single cell suspension from isografts were stimulated with PMA-ionomycin and we observed that increased TNFα, IFNγ, and CD107 were expressed by the CD4 and CD8 positive immune cells in the E0771 model, although significance was not reached.

## 5. Conclusions

In summary, we observed that NSC243928 treatment of the E0771 isograft tumor model led to significant changes in many immune markers that are desired to be employed in immuno-oncology (IO) therapies including PMN-MDSCs (neutrophils), NKT, and B1 cells. These changes were not observed in the NSC243928 treatment of 4T1 isograft tumor models. These results suggest that intrinsic properties of tumors will dictate the anti-tumor immune response triggered by NSC243928. E0771 represents a luminal B subtype and more precisely, the ERα^−^, ERβ ^+^, PR^+^, and ErbB2 ^+^ phenotypes, which respond to anti-estrogen treatments [7]. The 4T1 model represents a triple negative phenotype, which is not responsive to anti-estrogen treatments [6]. Because NSC243928 treatment led to significantly increased patrolling monocytes in both models, this suggests that it could be a more important immune cell type of NSC243928 action.

Clinical development for this novel drug like molecule may focus on testing whether NSC243928 treatment can increase the anti-tumor immune response in combination with anti-estrogen therapy for luminal B tumors and in combination with chemotherapy in triple negative breast cancer. Future studies are warranted to delineate the immune vs. non-immune effects of NSC243928 in vivo using mouse models.

## Figures and Tables

**Table 1:** Differentially expressed genes in the treated isografts. Gene function analysis using Pathway Studio^®^ (Elsevier, Inc. Amsterdam, Netherlands).

Condition	Upregulated Genes in Bold, Downregulated Genes in *Italic*	Function
Gene expression changes in NSC243928 treated E0771 isografts	**Tnfrsf21, Tspan9, Hacl1**	Immune activation [22]
	**Zfp953, Bnip5**	B-cell pathway associated genes [23]
	**Frem2, Celsr3, Mamdc2, Tm4sf1, Tnc, Gjb5**	Extracellular matrix regulation [24]
	**MIST1/Bhlha15**	Unfolded protein response [25]
	**Gpx3, Aox1**	Oxidation response [26]
	*Arg1, Irx30s, Rars2*	Arginine signaling [27]
	*Runx2, Wnt2b, Sox5, Irf5, Stap2, Pacsin1, Fnbp1l, Wrn, Nr2c1, Hspa1b, Bmp15*	Oncogenic pathways [28]
	*Ccl20, Cxcr5*	T-cell regulatory responses [29]
	*F12*	Extracellular matrix [30]
	*Dock10*	GTPase signaling [31]
Gene expression changes in NSC243928 treated 4T1 isografts	**Acacb, Cbarp, Has2**	Metabolism [32]
	**Ighv13-2, Cxcr1, Usp31, Cxcl2**	Immune activation [33]
	**Saa3, Defa39**	Extracellular matrix regulation [34]
	*Cidn7, Nog, Heg1, Plekhh1, Bicd1, Cox6a1, Flt4*	Oncogenic signaling [28]
	*Cd80, Siglece*	Immune suppression [35]
	*Top1mt*	Mitochondrial metabolism [36]
Common changes in both models	**Ccr8, Itgad, Cd3d, Foxp2**	Immune suppression [37]
	*Id4, Spast, Zgrf1, Ctsd, Grk2, Terc*	Oncogenic signaling [28]

## Data Availability

RNA sequencing data will be deposited with NCBI before publication.

## Supplementary Materials

The following supporting information can be downloaded at: https://www.mdpi.com/article/10.3390/cancers15051468/s1, Figure S1: Complete blots; Tables S1–S3.
