# Supplementary figures and images for: NSC243928 Treatment Induces Anti-Tumor Immune Response in Mouse Mammary Tumor Models
Source: Cancers (Basel). 2023 Feb 25;15(5):1468. doi: 10.3390/cancers15051468 (PMC10000927; doi:10.3390/cancers15051468)

E0771 cells

4T1 cells

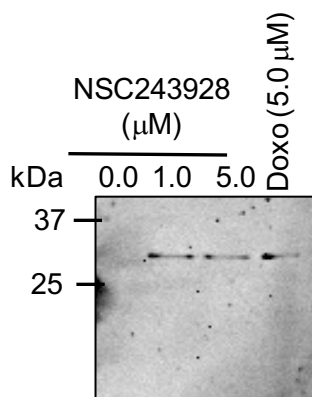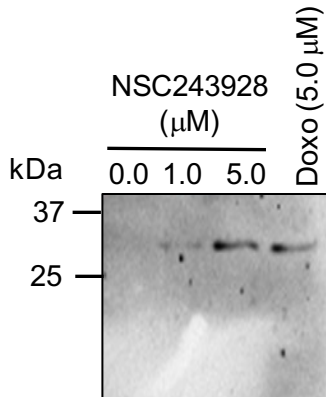

Blots Full

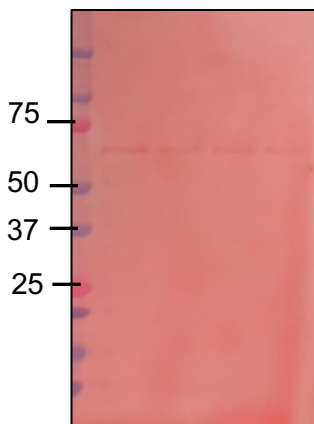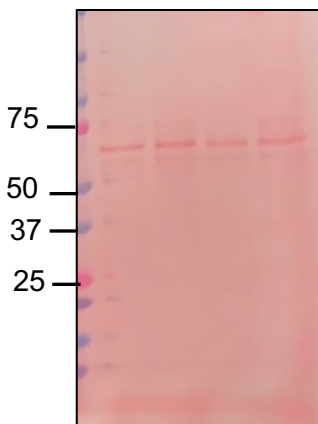

Ponceau membrane

Supplementary Figure S1: Complete blots

Supplement: Supplementary file 1 [file cancers-15-01468-s001.zip › Supplementary Figure S1.pdf]
